# A Global Path Planner for Safe Navigation of Autonomous Vehicles in Uncertain Environments

**DOI:** 10.3390/s20216103

**Published:** 2020-10-27

**Authors:** Mohammed Alharbi, Hassan A. Karimi

**Affiliations:** 1 Geoinformatics Laboratory, School of Computing and Information, University of Pittsburgh, Pittsburgh, PA 15260, USA; hkarimi@pitt.edu; 2 College of Computer Science and Engineering, Taibah University, Medina 42353, Saudi Arabia

**Keywords:** autonomous vehicles, sensors, sensor uncertainty, uncertainty assessment, uncertainty avoidance, autonomous navigation, path planner

## Abstract

Autonomous vehicles (AVs) are considered an emerging technology revolution. Planning paths that are safe to drive on contributes greatly to expediting AV adoption. However, the main barrier to this adoption is navigation under sensor uncertainty, with the understanding that there is no perfect sensing solution for all driving environments. In this paper, we propose a global safe path planner that analyzes sensor uncertainty and determines optimal paths. The path planner has two components: sensor analytics and path finder. The sensor analytics component combines the uncertainties of all sensors to evaluate the positioning and navigation performance of an AV at given locations and times. The path finder component then utilizes the acquired sensor performance and creates a weight based on safety for each road segment. The operation and quality of the proposed path finder are demonstrated through simulations. The simulation results reveal that the proposed safe path planner generates paths that significantly improve the navigation safety in complex dynamic environments when compared to the paths generated by conventional approaches.

## 1. Introduction

The development of fully autonomous vehicles (AVs) is currently inevitable. As modern vehicles, AVs have drawn an increasing amount of attention due to their potential to improve accessibility for individuals with disabilities and increase driver and passenger safety. Navigation is a key component of AVs, which requires a precise and accurate map for representing environmental features, namely roads, intersections, and traffic signals. Additionally, the functionality of AVs is dependent on the proprietary suites of sensors to continually collect data from their surroundings as they navigate the environment. The primary navigation sensors in AVs include the Global Navigation Satellite Systems (GNSSs), inertial measurement units (IMUs), cameras, and a light detection and ranging (LiDAR). Working collectively, these sensors perceive the motion and surroundings of AVs: the GNSS and IMU track the location and acceleration of the AV, and the other sensors detect the shape, distance, and velocity of nearby objects.

In addition to the localization task, AVs perform obstacle avoidance, object detection, and path planning, which together ensure safe navigation. The localization task estimates the location of the AV, whereas the aim of the obstacle avoidance task is to prevent collisions while navigating the environment. The objective of the path planning task is to determine a safe and efficient route from an initial point of interest to a requested destination with reference to a desired criterion, such as the shortest distance. With respect to the environment, path planning can be classified into two subgroups: global and local [1]. A global path planner computes a high-level trajectory leading to the destination, whereas a local path planner computes a suitable short-distance movement and maneuver for obstacle avoidance. While obstacle detection and avoidance helps in accident-free navigation, decisions are ultimately based on sensors that are not perfect in all environments [2]. Specifically, sensor performance varies in terms of accuracy, reliability, consistency, and availability. Sources of these variations are sensor uncertainties, which can generally be divided into two groups: static sources and dynamic sources. Static sources are fixed in location and do not vary over time. For instance, the GNSS is eminently susceptible to environmental conditions—e.g., GNSS receivers are unable to receive signals—while an AV drives through tunnels. However, dynamic sources, such as weather condition, vary with regard to their location, time, and severity. For example, when raining, the amount and intensity of rain and the droplet size substantially contribute to the level of image noise. Hence, AV navigation is highly influenced by these uncertainties. This influence leads to a high risk of traffic accidents, which could threaten the lives of passengers and other road users.

This work pays special attention to the global path planning in fully AVs considering the existence of sensor uncertainties. We propose a method for safe path planning, whose aim is to avoid areas where sensor uncertainties are expected to be high before a vehicle starts its journey and modifies a plan as changes occur. The proposed method utilizes a collection of fuzzy logics to model sensor uncertainties on road segments with the intention of predicting the uncertainties at a specific time. In addition, a cost function is developed to select a route with the lowest sensor uncertainty. The contributions of this paper include the design of a fuzzy logic–based uncertainty indicator and the development of a cost function for selecting routes with the lowest sensor uncertainty. The proposed method was evaluated using simulated data. The results demonstrate the effectiveness of the method and its capability in generating routes with least sensor uncertainties and thus ensuring safe navigation.

The rest of this paper is structured as follows. Section 2 discusses the related works. In Section 3, the proposed method and a brief review of the basics of the fuzzy logic reasoning technique are presented. In Section 4, the method is evaluated, and how the method can effectively determine safe routes is discussed. Section 5 concludes the key findings and discusses future research directions.

## 2. Related Works

Global path planners for AVs are primarily developed for the purpose of path finding and smoothing. A path finder searches for a set of waypoints to obtain the optimal route between two locations in a state space, whereas a path smoother enhances these waypoints by adding or subtracting sub-waypoints. The state space is typically configured as an occupancy grid or lattice. Path finding approaches can be further categorized based on their design paradigm into graph-based and sample-based planners. A graph-based planner uses either a deterministic search (e.g., Dijkstra’s algorithm [3]), which requires visitation of each node in the graph, or a heuristic search (e.g., A*’s algorithm [4]), which requires a priori knowledge to limit the number of nodes to be visited. A sample-based planner performs a random search within the state space to reduce the time complexity [5,6]. Heuristic and sample searches benefit local path planning due to their fast responses. A review of the algorithms that solve both global and local path planning problems for AVs is given in [7].

In regard to planning a global path in AVs, the literature contains different proposed optimization objectives. Among these objectives, several studies, such as [8], propose shortest-path planning. Note that the shortest path can be defined as either the traversed distance or time to travel between two points. Other studies have proposed path planning with energy efficiency as the optimization objective [9,10,11,12,13]. These planners aim to optimize energy in electric-powered vehicles because of the limitations in their battery capacity and the scarce availability of charging stations. Some studies propose path planners for resource constraints, including a time window [14], distance [15], or battlefield [16]. Furthermore, Simon [17] and Piazzi and Visioli [18] consider jerk minimization to enhance AV stability and passenger comfort along the planned path. Path planning that computes routes for AVs in the presence of sensor uncertainties is still an open problem.

Path planning that computes routes for AVs in the presence of uncertainties has received extensive attention among researchers since general-purpose planning algorithms are not applicable for AVs, especially in uncertain environments. The literature contains different planning approaches for uncertain environments. One approach incorporates the uncertainty as a belief space whose most general and systemic formulation is called the Partially Observed Markov Decision Process (POMDP) [19], where every possible belief (over states) is mapped into actions. This approach aims to obtain an optimal policy that achieves the maximum expected cumulative cost. Exact POMDP solutions are well-established for both finite [20] and infinite [21] horizon cases. In general, these planners consider a continuous belief space, requiring substantially complex computations and resulting in potential issues with scalability. Addressing scalability is the main fundamental idea that leads to the employment of sampling-based algorithms, including probabilistic roadmaps (PRM) [5] and rapidly-exploring random trees (RRT) [6]. An instance of the sampling-based planner includes the proposed belief roadmap planner for linear Gaussian systems [22]. Another approach leverages the Linear Quadratic Gaussian (LQG) methodology to construct a belief space. This approach allows for an estimation and a controller of linear systems with Gaussian noises [23]. The LQG-based planning approach involves a starting trajectory with two proposed common architectures. One architecture separates the process performed by the trajectory from the other linear control policies. Van den Berg, Abbeel, and Goldberg [24] use an RRT to generate candidate paths and evaluate the LQG performance over each one. Since their method aims to select the feasible path with respect to minimum cost having a bounded collision belief from an RRT, an optimal path might not be found. To address this, Bry and Roy [25] predict belief over trajectories of candidate paths using a local LQG, and then utilize an incremental sampling refinement to improve these paths. Other approaches perform an iterative distinct routine [26,27]. However, using simple distributions (e.g., Gaussian) to model uncertainty cannot precisely depict the belief of complicated systems, as in AVs’ navigating environment with sensor uncertainty. Furthermore, while these stochastic approaches can provide efficient solutions, the solutions are not always optimal. A planner that provides optimal solutions could only find a path in case one existed. With the stochastic approach, it is possible to have a situation where the planner would not succeed in finding a feasible path, even though one existed.

Navigation sensor uncertainties have been studied in various contexts. For instance, the impact of uncertainty on GNSS performance is discussed thoroughly in [28]. Roongpiboonsopit and Karimi [29] proposed an approach for predicting the quality of integrated GNSS. Hsu, Tokura, Kubo, Gu, and Kamijo [30] proposed an improved GNSS positioning approach that detects the GNSS inconsistency and excludes satellites with higher errors in urban canyon environments. Numerous studies [31,32,33] have also investigated the influence of weather conditions, such as rain and fog on LiDAR, radar, and camera sensors. Moreover, Alharbi and Karimi [2] have examined sensor uncertainties affecting the safety of road users during AV navigation; they proposed five algorithms to measure and highlight the navigation accuracy attainable in challenging environmental conditions.

Navigation sensor performance greatly depends on time and environment, as shown in [2]. Several approaches have proposed sensor fusion to reduce residuals and improve sensor stability [34,35]. Sensor fusion integrates the data of disparate sensors so that the fused data are more accurate than the data obtained from a single sensor. Although sensor fusion offers flexibility and shows effectiveness in handling uncertain data, it may lead to bias and divergence. The former comprises errors that account for the erroneous assumptions in the data fusion technique, whereas the latter occurs when the theoretical behavior of a data fusion model differs from its actual behavior. These issues impact the accuracy of data integration, which leads to limited safety considerations. Thus, there is a need for novel approaches that are capable of accurately detecting, analyzing, and avoiding sensor uncertainties.

In this paper, a new global path planning method, whose aim is to avoid challenging situations for navigation sensors in order to maximize AV safety, is proposed. We utilize multiple fuzzy logics to model challenging situations and obtain scores for their potential risk on each road segment. A cost function is used to calculate a weight based on sensor uncertainties for each road segment, which is used in Dijkstra’s algorithm to compute the optimal trajectory in the state space.

## 3. Method

**Figure 1:**
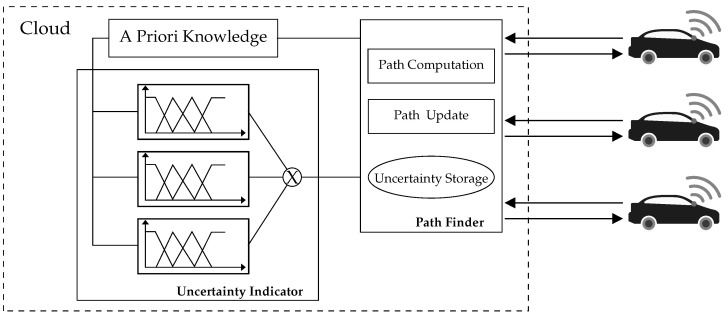
A proposed global safe path planner.

In this section, we first briefly review fuzzy logic and then discuss the architecture of the proposed method in the safe path planner. The proposed method includes a priori knowledge, an uncertainty indicator, and a path finder, as shown in Figure 1. We assume that the proposed path planning method is implemented in the cloud, so that the uncertainty indicator results can be shared with other AVs navigating in the same or similar environments, resulting in efficient resource usage. The priori knowledge consists of road networks, AV configurations, and sensor specifications. We assume these data are accurate, complete, updated, and sufficient to address all possible different environments for sensors.

### 3.1. Fuzzy Logic

**Figure 2:**
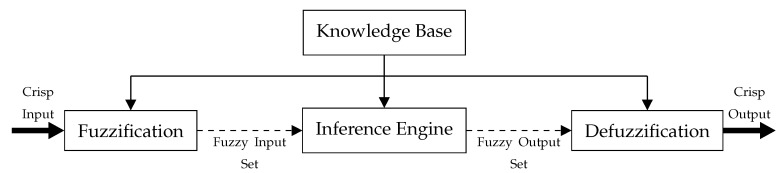
Fuzzy logic architecture.

Fuzzy logic [36] is a well-known representational and decision-making tool employed to handle variables that are imprecise and qualitatively uncertain. Given that the variables collected from the AV environment are mostly heterogeneous, fuzzy logic is employed to homogenize these variables into membership functions. Fuzzy inference rules are then applied to produce crisp outcomes. Figure 2 shows the fuzzy logic architecture, which has four primary phases. First, the fuzzification phase transforms the numerical input into a fuzzy set with a degree of membership, which is a real number in the interval [0, 1], where 0 and 1 are extreme cases of falsity and truth, respectively. Second, the inference engine phase determines the matching degree between the current fuzzy input and the if–then rules stored in the knowledge base. Last, the defuzzification phase generates a crisp outcome from the resultant fuzzy set obtained by the inference engine. The fuzzy sets are intended to reduce ambiguity. With this fuzzy logic approach, our objective is to determine the nature of an event in the AV environment. In particular, the approach enables the identification of whether an event influences the performance of a sensor.

### 3.2. Uncertainty Indicator

The uncertainty indicator measures the potential sensor failure on each road segment. We examine a scenario in which an AV is equipped with the GNSS, camera, and LiDAR sensors; their uncertainties are reported in [2]. As shown in Table 1, the sources of uncertainty for these sensors are classified into three categories. First, the road infrastructure, including tunnels, buildings, and trees, degrades the performance of the GNSS. For instance, tunnels and urban environments significantly reduce the quality of GNSS signals received from satellites. The road slope also reduces the coverage area of both the camera and LiDAR. Second, the capture time of these sensors may have a significant role in sensor performance. For instance, cameras are very sensitive to light, because pixel intensities are affected by changes in lighting conditions, which leads to poor performance of cameras at night. Third, extreme weather conditions, such as fog, rain, and snow, impair the sensor capability and mostly affect cameras and LiDAR.

To model the uncertainty, we use a combination of fuzzy logic controllers, where each controller represents a sensor. The outcomes are weighted and combined by the cost function (described in Section 3.3). These fuzzy logic controllers are hereafter called “fuzzy logic” or “*uncertainty indicators*” for the sake of simplicity. A fuzzy logic system is designed to qualitatively estimate risk when a specific sensor is considered. As Table 2 shows, the fuzzy logic system has ten unique input parameters. Overpass and underpass road structures are treated equally in this work since both types of structures prevent the GNSS from receiving signals. Tunnels and bridges are the most common underpass structure and overpass road structure, respectively. Thus, the *tunnel and bridge lengths* are inputs to the uncertainty indicator. Moreover, *buildings* and *trees*, obstructions to GNSS signal, are fed into the indicator. The building and tree lengths are defined as the average lengths of the blockages located near road segments. The uncertainty indicator also considers the *slope angle* of each road segment as an input. A road segment with no slope has an angle of 0${}^{\circ }$, whereas steep and inclined roads have positive and negative angles. The estimated *time* to arrive at a particular road segment is also input to determine the dim nighttime period, which is defined as the time between sunset and sunrise. In addition, four input parameters are considered to assess weather conditions—*visibility*, *snow depth*, *rainfall intensity*, and *fog*. Visibility and fog are normally evaluated by the meteorological optical range (MOR). The MOR is a measure of air transparency, which determines the longest distance, in kilometers, at which objects can be recognized by sensors. Snowfall is typically measured in centimeters. Rainfall intensity is expressed as the ratio of rainfall depth over a period of time (i.e., millimeters per hour).

Each input parameter is assigned a member function, thereby generating the fuzzy sets. The fuzzy set elements are divided based on the level of uncertainty into low-uncertainty elements and high-uncertainty elements. Low-uncertainty elements are *clear*, *covered*, and *bright*, while high-uncertainty elements are *occlusive*, *uncovered*, *dim*, *unclear*, and *obscured*. The following are the fuzzy sets used for determining sensor uncertainty:

- Tunnel/bridge, building, tree length— {*occlusive*, *clear*}
- Slope angle— {*uncovered*, *covered*}
- Time— {*dim*, *bright*}
- Visibility— {*unclear*, *clear*}
- Snow depth— {*obscured*, *clear*}
- Rainfall intensity— {*obscured*, *clear*}
- Fog— {*unclear*, *clear*}

**Figure 3:**
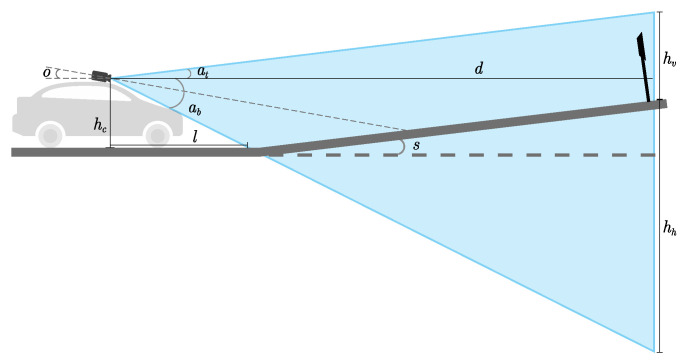
Vertical FOV of sensors.

From the sensor specifications and configurations, we derive the critical values, which define these parameter boundaries. For the camera and LiDAR fields of view (FOVs), we assume that at least one sensor with a clear distance, *d*, an aperture angle, *a*, and a certain optical angle, *o*, is mounted on the very front of the vehicle roof at a height, $h_{c}$. Figure 3 illustrates the vertical FOV of the sensors. As can be seen, the aperture angle *a* is separated by the horizontal sightline into the upper aperture angle $a_{t}$ and the lower aperture angle $a_{b}$. The blind spot length *l* is expressed as follows:

(1) $$l=h_{c}\tan\left(\frac{\pi }{2}-a_{b}\right),$$

By the properties of trigonometric functions, the occluded FOV height $h_{h}$ and visual height $h_{v}$ are expressed as

(2) $$h_{h}=\left(d-l\right)\left[\tan\left(a_{b}\right)+\tan\left(s\right)\right],$$

(3) $$h_{v}=d\left[\tan\left(a_{t}\right)+\tan\left(a_{b}\right)\right]-h_{h},$$

where *s* is the slope degree of the road segment. In the case of a steep path, the visual height must satisfy the following expression:

(4) $$h_{v}-h_{c}\geq 0.$$

By restricting the domain of the slope to the interval $\left(-\frac{\pi }{2},\;\frac{\pi }{2}\right)$ and solving for *s*, we obtain the fuzzy sets in Equation (5) for the slope parameter.

(5) $$f\left(s\right)=\left\{\begin{array}{cc} covered & -a_{b}\leq s\leq \tan^{-1}\left(\frac{d\tan\left(a\right)-h_{c}}{d-l}\right)-a_{b}\\ uncovered & -a_{b}\geq s\geq \tan^{-1}\left(\frac{d\tan\left(a\right)-h_{c}}{d-l}\right)-a_{b} \end{array}\right.$$

The S-shaped and Z-shaped membership functions [37] are used to generate the fuzzy variables and are given by Equations (6) and (7), respectively.

(6) $$f\left(x,a,b\right)=\left\{\begin{array}{cc} 0, & x\leq a\\ 2{\left(\frac{x-a}{b-a}\right)}^{2}, & a\leq x\leq \frac{a+b}{2}\\ 1-2{\left(\frac{x-a}{b-a}\right)}^{2}, & \frac{a+b}{2}\leq x\leq b\\ 1, & x\geq b \end{array}\right.$$

(7) $$f\left(x,a,b\right)=\left\{\begin{array}{cc} 1, & x\leq a\\ 1-2{\left(\frac{x-a}{b-a}\right)}^{2}, & a\leq x\leq \frac{a+b}{2}\\ 2{\left(\frac{x-a}{b-a}\right)}^{2}, & \frac{a+b}{2}\leq x\leq b\\ 0, & x\geq b \end{array}\right.$$

For a given universe of discourse *x*, a lower limit *a*, and upper limit *b*, the S-shaped and Z-shaped functions return the corresponding degrees of membership in the range [0,1]. One can easily observe that the S-shaped member function is the mirror image of the Z-shaped function. Going from the leftmost point *x* to the rightmost point in *x*, the output of the S-shaped function increases from 0 to 1, while that of the Z-shaped function declines from 1 to 0. These spline-based functions require the parameters *a* and *b* to establish the boundary of the sloped part of the line, with *a* < *b*.

Moreover, the fuzzy variables for the slope and time parameters utilize SZ-shaped and ZS-shaped membership functions defined in Equations (8) and (9), which are derived from Equations (6) and (7), respectively.

(8) $$f\left(x,a_{1:2},b_{1:2}\right)=\left\{\begin{array}{cc} 0, & a_{1}\geq x\geq b_{2}\\ 2{\left(\frac{x-a_{1}}{b_{1}-a_{1}}\right)}^{2}, & a_{1}\leq x\leq \frac{a_{1}+b_{1}}{2}\\ 1-2{\left(\frac{x-a_{1}}{b_{1}-a_{1}}\right)}^{2}, & \frac{a_{1}+b_{1}}{2}\leq x\leq b_{1}\\ 1-2{\left(\frac{x-a_{2}}{b_{2}-a_{2}}\right)}^{2}, & a_{2}\leq x\leq \frac{a_{2}+b_{2}}{2}\\ 2{\left(\frac{x-a_{2}}{b_{2}-a_{2}}\right)}^{2}, & \frac{a_{2}+b_{2}}{2}\leq x\leq b_{2}\\ 1, & b_{1}\leq x\leq a_{2} \end{array}\right.$$

(9) $$f\left(x,a_{1:2},b_{1:2}\right)=\left\{\begin{array}{cc} 1, & a_{1}\geq x\geq b_{2}\\ 1-2{\left(\frac{x-a_{1}}{b_{1}-a_{1}}\right)}^{2}, & a_{1}\leq x\leq \frac{a_{1}+b_{1}}{2}\\ 2{\left(\frac{x-a_{1}}{b_{1}-a_{1}}\right)}^{2}, & \frac{a_{1}+b_{1}}{2}\leq x\leq b_{1}\\ 2{\left(\frac{x-a_{2}}{b_{2}-a_{2}}\right)}^{2}, & a_{2}\leq x\leq \frac{a_{2}+b_{2}}{2}\\ 1-2{\left(\frac{x-a_{2}}{b_{2}-a_{2}}\right)}^{2}, & \frac{a_{2}+b_{2}}{2}\leq x\leq b_{2}\\ 0, & b_{1}\leq x\leq a_{2} \end{array}\right.$$

The output parameter for each fuzzy logic controller is called *risk*. Risk is defined as the probability that the sensor is exposed to uncertainty. The higher the risk associated with a road segment, the more uncertainty the sensor has. We assume that no external factors, including dynamic objects such as pedestrians and other vehicles, influence the sensor reading quality. The fuzzy set for determining risk consists of the variables *low* and *high*. A triangular membership function expresses the fuzzy outputs, as given in Equation (10).

(10) $$f\left(x,a,b,c\right)=\max\left(\min\left(\frac{x-a}{b-a},\frac{c-x}{c-b}\right),0\right)$$

where *x* is the universe of discourse; *a* and *c* are the lower and upper limits where the degree of membership is 0, respectively; *b* is the value where the degree of membership is 1, —i.e., a<b<c.

The fuzzy IF-THEN rules evaluated by the inference engine are given in Table 3. Broadly speaking, the fuzzy rules are as follows:

Rule 1: If a parameter value is *clear*, *covered*, and *bright*, then the *risk* is low.
Rule 2: If a parameter value is *occlusive*, *uncovered*, *dim*, *unclear*, or *obscured*, then the *risk* is high.

### 3.3. Path Finder

The path finder aims to select a route with the minimum likelihood of sensor failure. The path finder comprises three tasks: path computation, path update, and uncertainty storage. The following subsections discuss these tasks in detail.

#### 3.3.1. Path Computation

At the highest level, path computation aims to select a route between an AV’s current position and the requested destination. This problem is represented as a graph. Road networks are typically represented as a directed graph, G=(V,E,w), whose edge weights, w:E→N, are equivalent to the traversal cost for a road segment, edge(u,v)∈E. The traversal cost is defined as the ratio of sensors mounted in a vehicle to their risk scores reported by the uncertainty indicator. Therefore, the task of finding the optimal route can be expressed as a shortest-path problem and solved using classical algorithms, such as Dijkstra’s algorithm [3] and A*’s algorithm [4]. In this work, Dijkstra’s algorithm is adopted because it explores all vertices in the graph. Note that the performance of the algorithm, which is exponential and not efficient for very large networks, is not addressed in this work.

The cost function has the form

(11) $$\min w\left(v\right)=g\left(v\right)+\alpha \;d\left(v\right),$$

where *v* is the current vertex, $g\left(v\right)$ is the sensor uncertainty, $d\left(v\right)$ is the Euclidean distance between the current vertex and the destination, and α is a hyperparameter that controls the distance weight. The value of α needs to be determined; according to our simulation a value near 0.5 would reduce the impact of distance on the overall cost. The Euclidean distance aims to penalize any deviations from the reference path, which is the straight path from the current location to the target destination. In this way, we reduce the uncertainty level to an acceptable degree while maintaining a reduced path length.

The sensor uncertainty function can be expressed as

(12) $$g\left(v\right)=M^{-1}\sum_{i\in M}{\rho_{i}s}_{i}+\varphi \left(s_{i}\right),$$

where M is the number of sensors, $\rho_{i}$ represents how much the AV subsystems rely on the $i^{th}$ sensor $\left(0<\rho_{i}\leq 1\right)$, and $s_{i}$ is the risk indicator score, which ranges between 0 and 1. $\varphi \left(s\right)$ is a penalty term used to maximize the number of high-quality sensors, which is given by

(13) $$\varphi \left(s_{i}\right)=\left\{\begin{array}{cc} 1, & s_{i}>0.5\\ 0, & s_{i}\leq 0.5 \end{array}\right.$$

#### 3.3.2. Path Update

A decision based on dynamic uncertainty evaluation requires frequent updates. Without these updates, AVs might be exposed to unknown uncertainties. For this, a path update algorithm is used to monitor the generated path for any change. When a major change occurs to road segment uncertainty, such that the risk score switches from high to low or vice versa, the path update algorithm requests the current path to be recomputed. To make this process fast and thus meet the real-time requirement of AVs, only a few road segments ahead of the current are evaluated. Algorithm 1 shows the path update process.
**Algorithm 1:** Path Update Algorithm
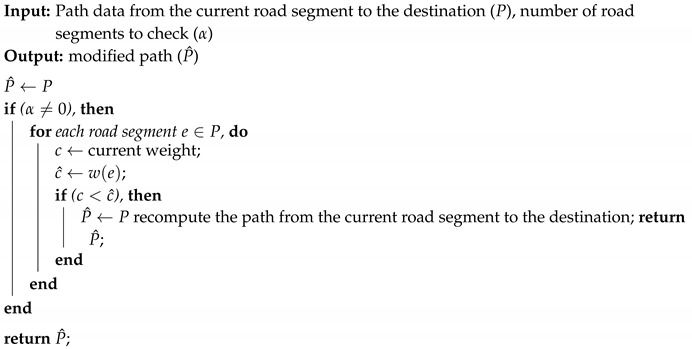


#### 3.3.3. Uncertainty Storage

AV subsystems could exclude sensors with high potential risk to improve their performance. We establish an uncertainty storage to maintain the sensors’ risk scores for each road segment, which is selected on the generated route. By querying these scores, AV subsystems can examine the quality of each sensor in their current situation to ensure that they can make more accurate decisions in regard to sensors’ reliability. The uncertainty storage is updated whenever a path re-computation is performed. In addition, uncertainty storage offers easy and more efficient source management for AVs that have identical sensor specifications and configurations. Since the proposed path planner is implemented in the cloud platform, all AVs can utilize the precomputed uncertainty scores. Hence, with the cloud-based path planner, AVs would benefit from the cloud computing power in processing and maintaining less data.

## 4. Evaluation and Discussion

**Figure 4:**
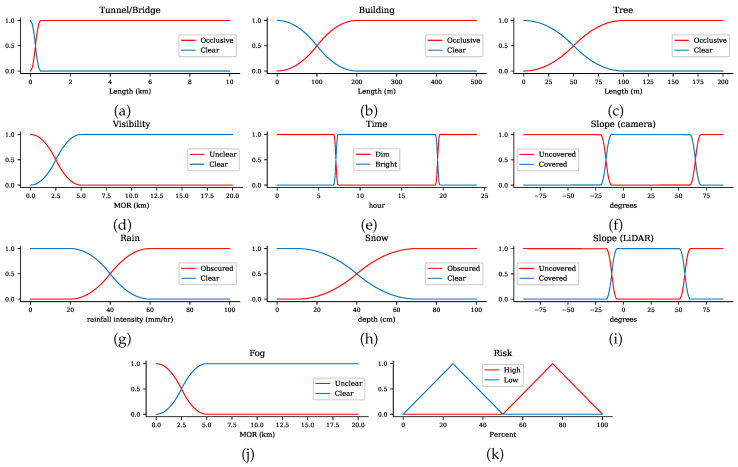
Membership functions for the uncertainty indicator.

The proposed path planner was implemented in Python. For fuzzy logic implementation, the Fuzzy Logic SciKit was adopted since it includes all types of fuzzy membership functions. Due to the difficulty of obtaining an AV with all tested sensors, our proposed method was evaluated by using simulated data. For sensor uncertainty sources, we considered buildings, trees, tunnels, road slope, time, visibility, and weather conditions. The uncertainty thresholds for each source used in our simulation are given in Table 4. For instance, the maximum tunnel and bridge lengths tolerated by sensors was 500 m. The night time was set between 19:35 (sunset time) and 7:20 (sunrise time). The road slope thresholds were computed using Equation (5), resulting in [−10.7, 70.1] and [−5.56, 60.59] for the camera and LiDAR, respectively. Table 4 reveals the remaining parameters, including sensor specifications. The membership functions of the uncertainty indicator are presented in Figure 4. A simulated grid road network was constructed for evaluating our proposed path planner method. In the road network, we assigned arbitrary uncertainty sources to road segments. We evaluated the performance of the proposed method against a conventional planner, which simply optimized the route length. The performance was evaluated in terms of: (1) the number of uncertainty sources in the computed path and (2) the ability to omit road segments that have only sensors with high risk scores.

**Figure 5:**
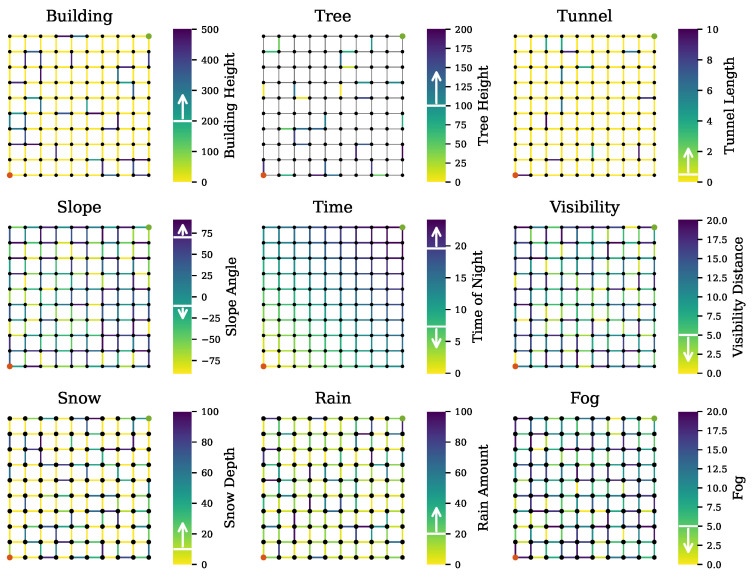
Simulated road network with uncertainty sources. The vertical color bars denote the parameters’ values and the arrow indicates the risk direction.

Figure 5 shows the simulated road networks annotated with the assigned uncertainties. The vertical color bars denote the parameters’ values. The arrow shown in the color bars indicates the risk direction. The uncertainty sources considered in this evaluation can be classified as dynamic or static. The static sources were buildings, trees, and tunnels, and the dynamic sources were slope, time, visibility, and weather conditions. The road network in Figure 5 (middle) shows the arrival time, which was calculated based on velocity and assumed to be constant, and road segment length. In the presence of these uncertainties, the route between the lower left node (in red) and the upper right node (in green) was computed.

The proposed path planner was thoroughly tested in three different scenarios. The first scenario was that of equal-weighted sensors. In this case, the reliance term, $\rho_{i}$, was equal to 1 for all sensors. The second scenario concerned preferable sensors, among which only one sensor had a higher reliance weight. If sensor *X* has a higher reliance weight than the others, it is called preferable. In the third scenario, two out of three sensors were assigned equal weights $\left(\rho_{i}=1\right)$, and one was assigned a lower weight $\left(\rho_{i}=0.5\right)$. Table 5 shows the weights of the sensors contributing to each scenario. All scenarios utilized the same road network with identical setups as mentioned above. We checked the proposed path planner with different distance weights α and in the presence or absence of the penalty term $\varphi (s_{i})$ for the number of low-quality sensors. The outcomes revealed that the proposed path planner performs better than the conventional planner in all scenarios.

**Figure 6:**
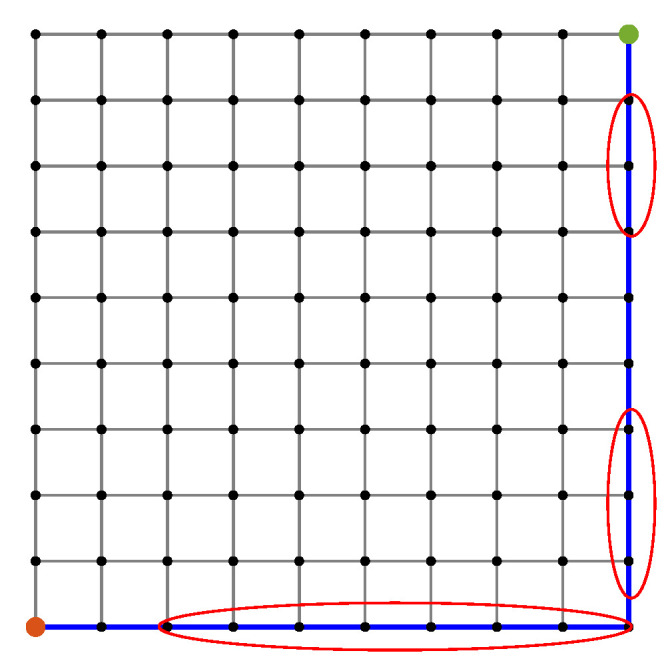
Path planner using the conventional approach. Red circles mark instances of the road segments with multiple high-risk sensors.

**Figure 7:**
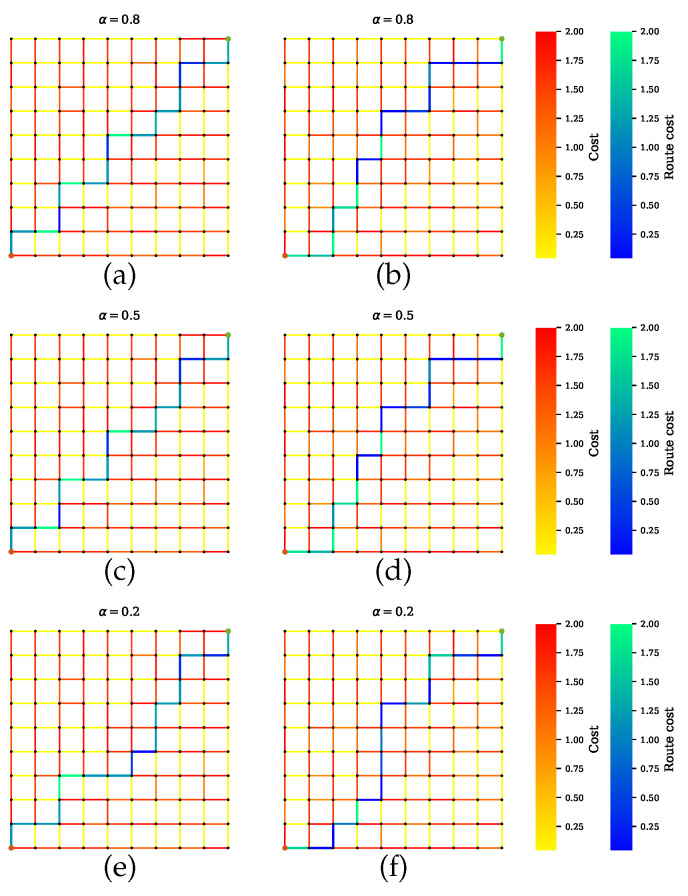
Path planner for scenario A that balances all sensors’ performances during planning. (**a**,**c**,**e**) Proposed method with a penalty term. The planner finds paths with maximum number of sensors and least combined amount of uncertainty. Increasing the value of α leads to a shorter path. The solutions in (**b**,**d**,**f**) exclude the penalty term, so the paths can indicate a lower uncertainty, but the joint sensor quality is not guaranteed.

Figure 6 shows the path selected by the conventional path planner, which executes Dijkstra’s algorithm with the shortest length as a cost function. The generated path has certain road segments with multiple high-risk sensors, which are not safe. This is because the conventional approach does not take into account the sensor performance for finding optimal routes. As it is shown, some road segments with low uncertainty are parts of the shortest path. On the other hand, Figure 7 shows our proposed method for Scenario A, which balances performances by all sensors. The proposed method successfully avoids encountering uncertainty. By penalizing the low-quality sensors, as shown in Figure 7a,c,d, the generated path includes the maximum number of reliable sensors and thus is a safe route. However, disregarding the quality term, as shown in Figure 7b,d,f, leads to the inclusion of high-risk road segments along the route. Furthermore, increasing the distance weight α can force a planner to select one road segment over another road segment regardless of the uncertainty.

**Figure 8:**
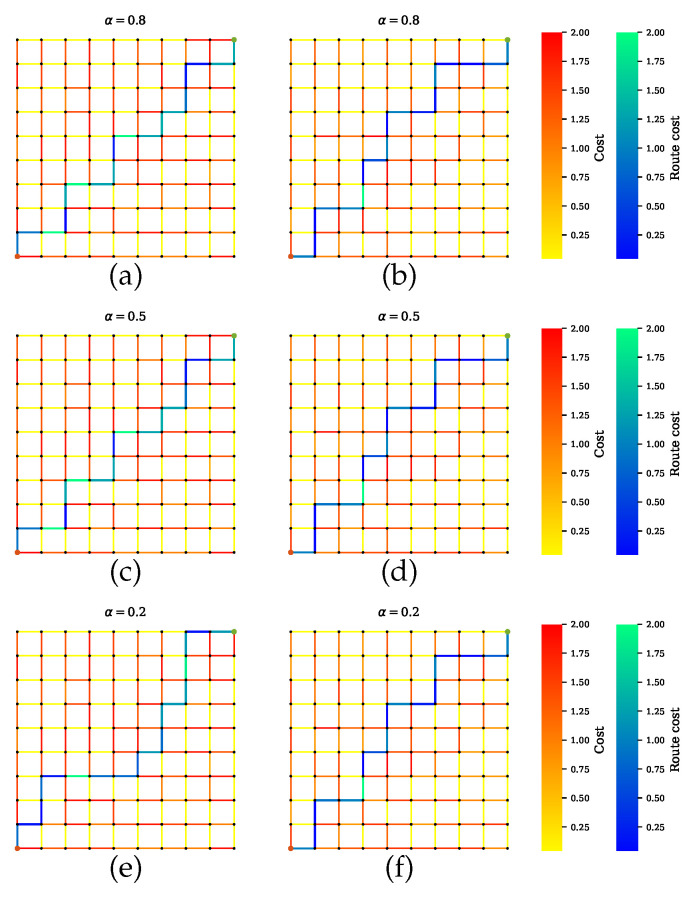
Path planning for scenario B.1 when GNSS is preferable over other sensors. (**a**,**c**,**e**) Proposed method with a penalty term. The computed paths greatly function with GNSS. Some trajectories with high risk for GNSS include in the paths due to the penalty term effect. (**b**,**d**,**f**) Proposed method without a penalty term effect. Trajectories with high risk for GNSS are filter out.

**Figure 9:**
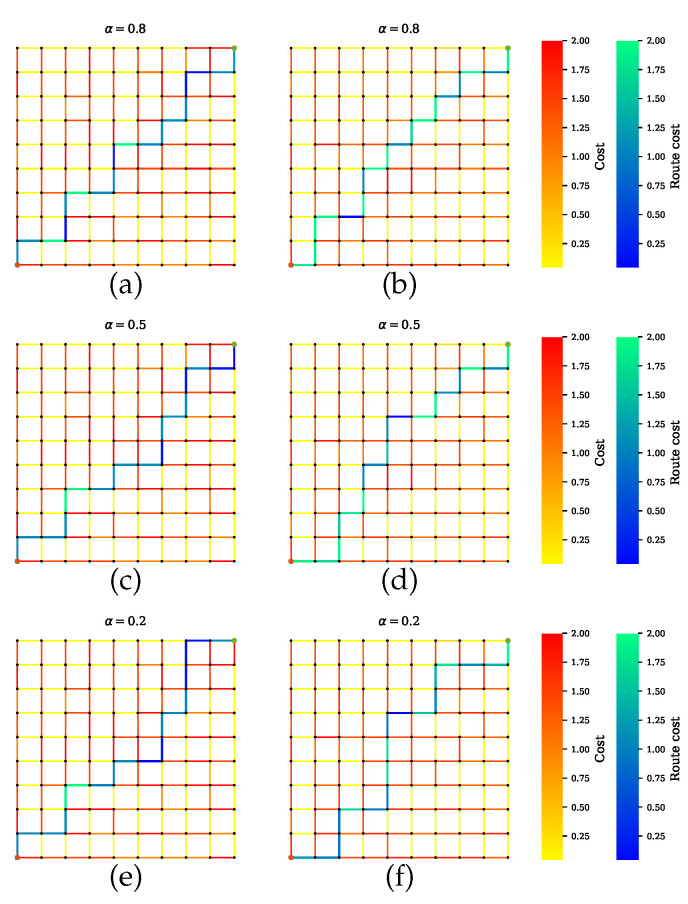
Path planning for scenario B.2 when camera is preferable over other sensors. (**a**,**c**,**e**) Proposed method with a penalty term. (**b**,**d**,**f**) Proposed method without a penalty term.

**Figure 10:**
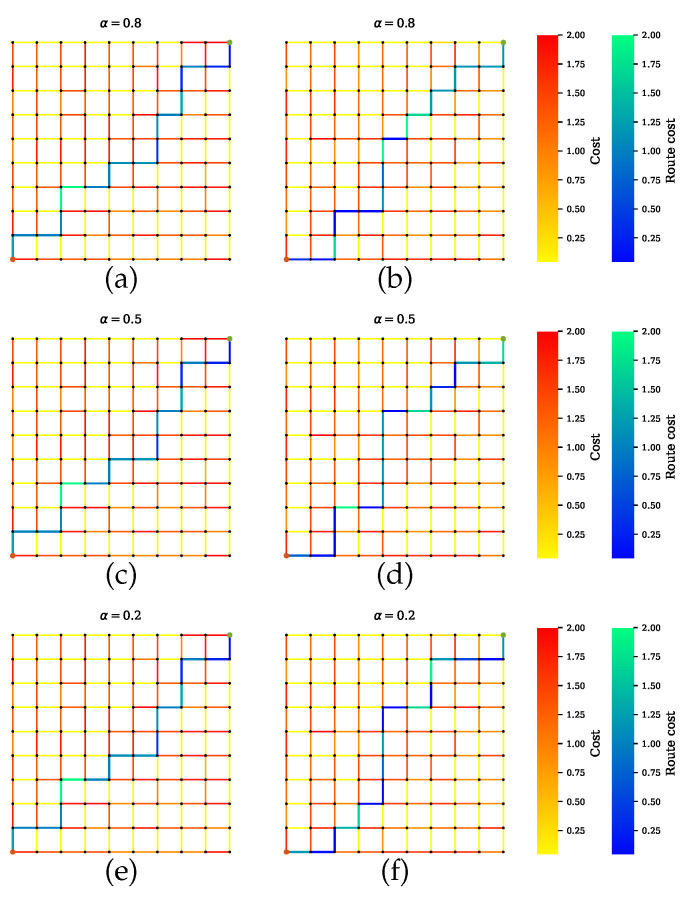
Path planning for scenario B.3 when LiDAR is preferable over other sensors. (**a**,**c**,**e**) Proposed method with a penalty term. (**b**,**d**,**f**) Proposed method without a penalty term.

Scenario B shows the simulations where AVs rely on only one sensor. Figure 8 presents the GNSS preferable path where the planner disregards the road segments with tunnels, which have high uncertainties. Likewise, a precise path is computed for cameras and LiDAR, as shown in Figure 9 and Figure 10, respectively. From Figure 8b,d,f, Figure 9b,d,f and Figure 10b,d,f, it is obvious that neglecting the penalty term for the sensors with high uncertainty results in only one sensor being utilized.

**Figure 11:**
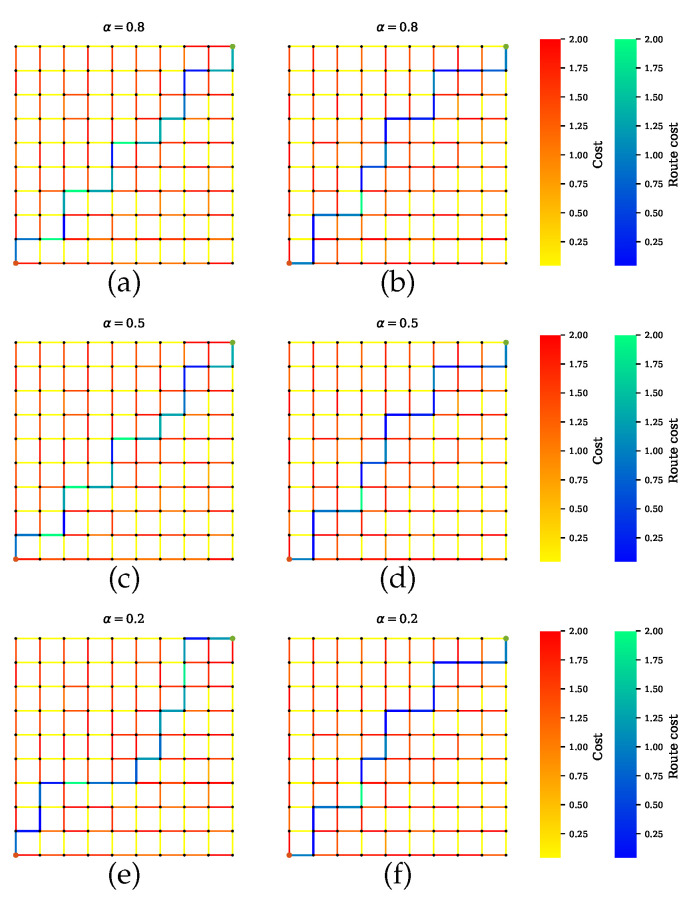
Path planning for scenario C.1 when GNSS and camera are preferable sensors. (**a**,**c**,**e**) Proposed method with a penalty term. (**b**,**d**,**f**) Proposed method without a penalty term.

**Figure 12:**
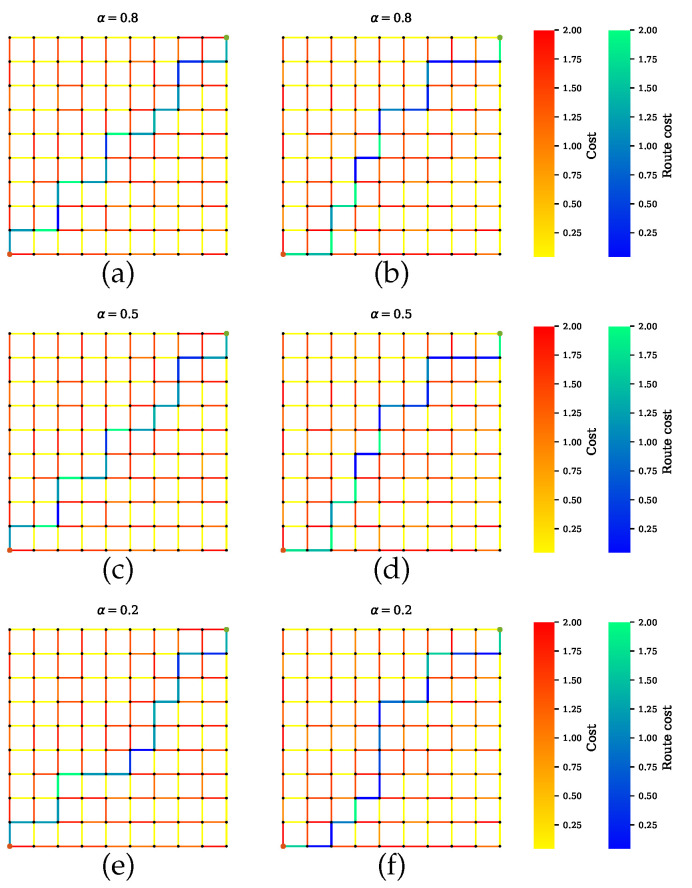
Path planning for scenario C.2 when GNSS and LiDAR are preferable sensors. (**a**,**c**,**e**) Proposed method with a penalty term. (**b**,**d**,**f**) Proposed method without a penalty term.

**Figure 13:**
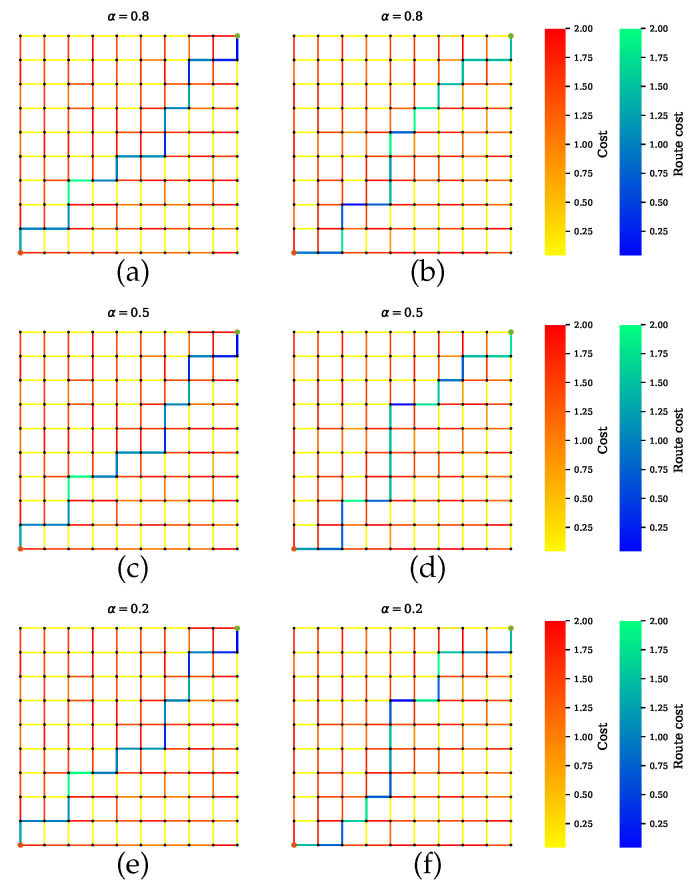
Path planning for scenario C.3 when camera and LiDAR are preferable sensors. (**a**,**c**,**e**) Proposed method with a penalty term. (**b**,**d**,**f**) Proposed method without a penalty term.

Scenario C shows the simulations where AVs rely on all but one sensor. Figure 11, Figure 12 and Figure 13 show the computed path for the GNSS, camera, and LiDAR, which the planner disregards. The proposed planner prioritizes the remaining sensors and successfully calculates the low-uncertainty path. This is illustrated in Figure 13 where the camera and LiDAR are the preferred sensors over GNSS. Thus, the computed path includes some road segments with uncertainties (e.g., tunnels) that affect the performance of GNSS only.

**Figure 14:**
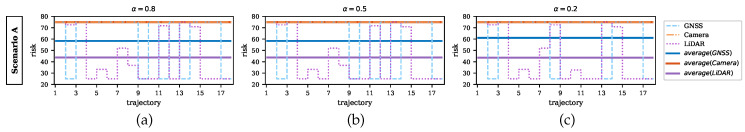
Risk of the planned path for scenario A where sensors’ performances are balanced. Horizontal lines depict the average of sensors’ risk scores. (**a**–**c**) show different distance weight trails—i.e., α=0.8, α=0.5, and α=0.2, respectively. The average of sensors’ risk scores is almost equal among all trails. However, as the distance weight is very small, the planned paths demonstrate high sensor quality since the planner reduces the distance power and avoids trajectories with no perfect sensors.

**Figure 15:**
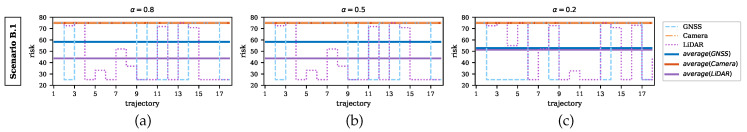
Risk of the planned path for scenario B.1 where GNSS is preferable over other sensors. Horizontal lines depict the average of sensors’ risk scores. (**a**–**c**) show different distance weight trails—i.e., α=0.8, α=0.5, and α=0.2, respectively. The GNSS performance on average is similar among all trails, with slight improvement in (c). LiDAR’s performance expectedly drops in (c) as there is no constraint on its performance. With lower distance weight, the planned paths demonstrate high sensor quality since they avoid trajectories with no perfect sensors.

**Figure 16:**
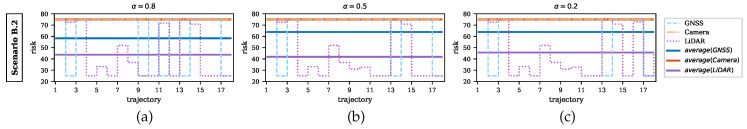
Risk of the planned path for scenario B.2 where camera is preferable over other sensors. Horizontal lines depict the average of sensors’ risk scores. (**a**–**c**) show different distance weight trails—i.e., α=0.8, α=0.5, and α=0.2, respectively. The camera’s performance on average is identical among all trails since cameras are vulnerable to many uncertainty sources in the environment. The GNSS’s performance expectedly drops in (b) and (c) as there is no constraint on its performance. As the distance weight is very small, the planned paths demonstrate high sensor quality since they avoid trajectories with no perfect sensors.

**Figure 17:**
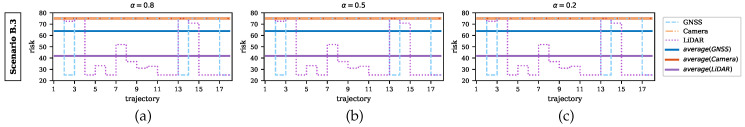
Risk of the planned path for scenario B.3 where LiDAR is preferable over other sensors. Horizontal lines depict the average of sensor risk scores. (**a**–**c**) show different distance weight trails—i.e., α=0.8, α=0.5, and α=0.2, respectively. All sensors have an identical performance among all trails since the best planned path is not impacted by the distance constraint.

**Figure 18:**
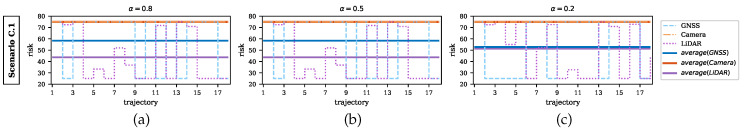
Risk of the planned path for scenario C.1 where LiDAR is neglected. Horizontal lines depict the average of sensors’ risk scores. (**a**–**c**) show different distance weight trails—i.e., α=0.8, α=0.5, and α=0.2, respectively. (a) and (b) reveal no change in sensor performances. In (c), the performance is improved for GNSS and deteriorated for LiDAR, which is the targeted objective in this scenario.

**Figure 19:**
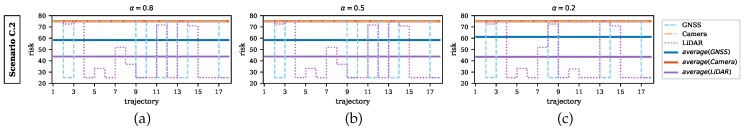
Risk of the planned path for scenario C.2 where camera is neglected. Horizontal lines depict the average of sensors’ risk scores. (**a**–**c**) show different distance weight trails—i.e., α=0.8, α=0.5, and α=0.2, respectively. These trails indicate an improvement in the sensor quality when the distance weight reaches 0.2. Additionally, the performances of GNSS and LiDAR are improved in some trajectories but remain steady on average.

**Figure 20:**
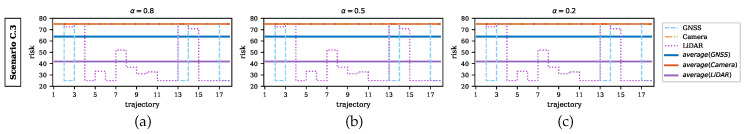
Risk of the planned path for scenario C.3 where GNSS is neglected. Horizontal lines depict the average of sensors’ risk scores. (**a**–**c**) show different distance weight trails—i.e., α=0.8, α=0.5, and α=0.2, respectively. These trails indicate no change in sensor uncertainties since the best planned path is not impacted by the distance constraint.

Furthermore, quantitative analysis is performed in terms of measuring the risk over the whole route. The risk score is calculated from the uncertainty storage. A high risk score indicates that the route is undesirable for navigation, as it poses high sensor uncertainty. This analysis provides further verification of the feasibility of the computed path for each scenario. The results per scenario with different distance weights are demonstrated in Figure 14, Figure 15, Figure 16, Figure 17, Figure 18, Figure 19 and Figure 20. The average of risks is drawn as horizontal lines. All paths computed by the proposed path planner optimize the objective in each scenario. For instance, Figure 14 reveals the risk of the planned path which is contributed equally by each sensors. The planned path indicates a trade-off between distance and risk. As the distance weight decreases, paths have lower uncertainty, and the planner has a better chance to maximize the sensor quality. Figure 17 and Figure 20 show cases where there is no impact by the distance constraint on risk scores since the best planned path is very close to the reference path. Thus, the distance weight requires careful tuning. We recommend a value of 0.5 for the distance weight, because this value balances the route length and the likelihood of encountering uncertainty. This objective is realistic because a planned route should not deviate far from a reference route. Moreover, with knowledge of the uncertainty scores for each road segment, AVs can feed these scores into their systems to better handle uncertain environments with the goal of achieving safe navigation.

## 5. Conclusions and Future Research

In this paper, a global path planner method with the goal of finding routes with minimum sensor uncertainties is proposed. To satisfy the requirement of real-time navigation, the proposed method quantifies sensor uncertainty using fuzzy logic systems. More specifically, the proposed path planner weighs road segments with risk scores to compute a global path with the objective of not deviating far from the shortest path between the same pair of origin and destination locations. The simulation results demonstrate that the proposed path planner method effectively enhances path computation in terms of least overall combined sensor uncertainties.

Although path planning under uncertainty in AVs is a well-researched area, to the best of our knowledge, this work is the first attempt to design and formulate a path planner that considers detecting, analyzing, and avoiding sensor uncertainties’ sources in structured environments. Various parameters are determined via simulation experiments, including the threshold of each uncertainty source and cost function parameters. However, more work is required to effectively optimize these parameters. Additionally, in real scenarios, AVs would have their own high-definition maps, which include all road characteristics and surroundings, stored in a database. These data could be a priori knowledge for our proposed approach. In this work, we assume that all data in this database are accurate, complete, updated, and sufficient to address all possible different challenges in the environments. Thus, one future research direction is to manage uncertainty in this database. Furthermore, the key objective of this work is to address safety in navigation by considering the subset of sensors that can provide a low overall level of uncertainty. Future directions also include optimizing ride/route smoothness that enables maintaining stability and comfort along the route.

## Figures and Tables

**Table 1:** Sources of uncertainty for sensors.

Sensor	Road Infrastructure	Time	Weather
**GNSS**	Tunnels/bridges		
	High-rise buildings		
	Dense trees		
**Camera**	Slope	Night	Fog Rain Snow visibility
**LiDAR**			

**Table 2:** Input parameters of fuzzy logic.

Sensor	Parameter	Unit
**GNSS**	Tunnel/bridge length	km
	Building length	m
	Tree length	m
**Camera**	Slope angle	radians
	Time	HH:mm
	Visibility (MOR)	km
**LiDAR**	Slope angle	radians
	Snow depth	cm
	Rainfall intensity	mm/h
	Fog (MOR)	km

**Table 3:** Fuzzy rules (risks).

Risk	Parameter	Fuzzy Set
	Tunnel/bridge length	occlusive
	Building length	occlusive
	Tree length	occlusive
	Slope angle	uncovered
**High**	Time	dim
	Visibility	unclear
	Snow depth	obscured
	Rainfall intensity	obscured
	Fog	unclear
	Tunnel/bridge length	clear
	Building length	clear
	Tree length	clear
	Slope angle	covered
**Low**	Time	bright
	Visibility	clear
	Snow depth	clear
	Rainfall intensity	clear
	Fog	clear

**Table 4:** Simulation parameters.

Paremeter	Value	Unit		Sensors Specifications		
Tunnel/bridge length	0.5	km	**Sensor**	**Paremeter**	**Value**	**Unit**
High-rise building length	200	m	**Camera**	optical angle	10	deg.
Dense tree length	100	m		aperture angle	80	deg.
Time	07:20	HH:mm		observable distance	120	m
	07:20	HH:mm		camera height	1.9	m
Visibility	5	km	**LiDAR**	optical angle	5	deg.
Snow depth	10	cm		vertical FOV	64	deg.
Rainfall intensity	20	mm/h		distance range	200	m
Fog	5	km		LiDAR height	1.9	m
Risk percent	50	percent				

**Table 5:** Simulation scenario parameters.

Scenario	$\rho_{\mathrm{GNSS}}$	$\rho_{\mathrm{camera}}$	$\rho_{\mathrm{lidar}}$
**A**	1	1	1
**B.1**	1	0.5	0.5
**B.2**	0.5	1	0.5
**B.3**	0.5	0.5	1
**C.1**	1	1	0.5
**C.2**	1	0.5	1
**C.3**	0.5	1	1
